# A single sample GnRHa stimulation test in the diagnosis of precocious puberty

**DOI:** 10.1186/1687-9856-2012-23

**Published:** 2012-07-18

**Authors:** Parvin Yazdani, Yuezhen Lin, Vandana Raman, Morey Haymond

**Affiliations:** 1 Pediatric Endocrinology, Baylor College of Medicine, Houston, 77024, TX, USA; 2 University of Utah, Salt Lake City, UT, USA; 3 Baylor College of Medicine, Children’s Nutritional Research Center, Houston, TX, USA

**Keywords:** Central precocious puberty, Luteinizing hormone, Gonadotropin releasing hormone analogue

## Abstract

**Context:**

Gonadotropin-releasing hormone (GnRH) has been the standard test for diagnosing central precocious puberty. Because GnRH is no longer available, GnRH analogues (GnRHa) are now used. Random LH concentration, measured by the third-generation immunochemiluminometric assay, is a useful screening tool for central precocious puberty. However, GnRHa stimulation test should be considered, when a basal LH measurement is inconclusive. However optimal sampling times for luteinizing hormone (LH) have yet to be established.

**Purpose:**

To determine the appropriate sampling time for LH post leuprolide challenge.

**Methods:**

A retrospective analysis of multi-sample GnRHa stimulation tests performed in 155 children (aged 1–9 years) referred for precocious puberty to Texas Children’s Hospital.

After 20 mcg/kg of SQ leuprolide acetate, samples were obtained at 0, 1, 3, and 6 hours.

**Results:**

Of 71 children with clinical evidence of central precocious puberty, fifty nine children had a peak LH >5 mIU/mL. 52 (88%) of these responders had positive responses at 1 hour (95% CI is 80–96%), whereas all 59 children (100%) had a peak LH response >5 mIU/mL at 3 hours (95% CI is 94-100%), P = 0.005.

**Conclusions:**

A single serum LH sample collected 3 hours post GnRHa challenge is the optimal sample to establish the diagnosis of central precocious puberty.

## Background

Central precocious puberty is the early onset of pubertal development as a result of gonadotropin release by the pituitary gland. Precocious puberty in a child can be associated with adverse consequences including compromised final adult height and psychosocial problems. Establishing the diagnosis of central precocious puberty requires documenting pubertal physical findings and measuring luteinizing hormone (LH) concentration, which is the key biochemical assessment of pubertal status. Gonadotropin-releasing hormone (GnRH)-stimulated plasma LH concentrations have been the mainstay for establishing the diagnosis of precocious puberty, but it is no longer available in the United States. GnRH analogue (leuprolide acetate) administered subcutaneously is a suitable substitute for GnRH in the diagnosis of central precocious puberty [1-5]. Ibanez et al. reported that a peak serum LH response >8 IU/L occurred in patients with progressive puberty and in patients with Tanner stage II puberty 3 hours post leuprolide acetate challenge [3]. The LH concentrations declined progressively from 3 to 6 hours post-stimulation. In patients with non-progressive puberty and in pre-pubertal controls, the LH peak occurred between 3 and 6 hours after injection [3].

Rosenfield et al. measured gonadotropin levels at 0, 2, 4, 8, 16, and 24 hours post leuprolide acetate injection in a dose–response study comparing acute hormonal responses of the GnRHa leuprolide acetate to GnRH in 15 women and 15 men [6]. They reported peak plasma LH concentration at 1 and 4 hours in men and women, respectively. Other investigators using alternative sampling times demonstrated peak sample at 1 hour following leuprolide administration [2]. A previous study by Houk et al. demonstrated that a single LH measurement obtained 30 minutes post GnRHa stimulation provided adequate information to ascertain pubertal status in girls. However, they examined GnRHa stimulation testing with single 30-minute post-stimulus gonadotropin measurements, and none of the patients in their study had slowly progressive puberty [7]. Thus, despite the evidence of the efficacy of subcutaneous leuprolide acetate in the diagnosis of central precocious puberty, the optimal sampling times for LH post leuprolide challenge has not yet been determined based on the published results. The objective of our study is to re-evaluate optimal sampling time for LH post leuprolide acetate challenge in a cohort of patients presenting with signs of early pubertal development.

## Materials and Methods

We conducted a retrospective analysis of the results of leuprolide acetate stimulation tests in children referred for possible precocious puberty to Texas Children’s Hospital from January 2003-December 2006. During this time, we utilized a multiple sampling protocol as described below.

Children of both genders and all ethnicities (aged 1–9 yrs) were identified who had undergone this multi-sample leuprolide acetate (20 mcg/kg SQ) stimulation tests for the suspected diagnosis of central precocious puberty [2]. Serum luteinizing hormone (LH) and follicle stimulating hormone (FSH) concentrations were measured at 0, 1, 3 and 6 h post-injection. Serum estradiol and testosterone concentrations were measured at 0 and 6 h. Of the 155 subjects identified, one subject did not have a blood sample taken at 6 hours; the data from this subject was included since the analysis of the overall data including or excluding these data did not affect the results or conclusions and the primary comparisons were between 1 and 3-hour samples.

The diagnosis of central precocious puberty was established based on the clinical history of onset of pubertal changes (girls <8y, boys <9y), physical examination suggesting puberty based on Tanner-stage breast and pubic hair development in girls, testicular volume in boys, growth velocity over at least 6 months and bone age.

Based on these criteria and a follow up of at least 6 months with the exception of a few cases in which thelarche resolved after 4 months, the subjects were divided into three groups: A. Non-progressive puberty with thelarche, B. Non-progressive puberty with adrenarche, and C. Central precocious puberty.

### Gonadotropin and sex steroid assays

The serum LH and FSH concentrations were analyzed at our clinical laboratory using the ADVIA Centaur immunoanalyzer (a two-site sandwich immunoassay) and direct chemiluminometric technique (ICMA, third-generation assay). The sensitivity of the FSH and LH assays were 0.3 mIU/mL and 0.07 mIU/mL, respectively. The published per cent coefficient of variation for replicate analysis were <4% for both assays in the 0.3-200 mIU/ml for FSH and 0.07-200 mIU/mL for LH, and the precision accuracy of assay was validated according to CAP laboratory accreditation standards. Serum estradiol and testosterone were measured by LCMS at Esoterix, Calabasas Laboratory.

### Statistical analysis

All data are provided as mean ± SEM. An LH value < 0.1 mIU/mL was assumed to be 0.1mIU/ml for purposes of calculations. The generalized estimating equations method for the binomial distribution and logit link function (SPSS 18.0) was used to estimate and compare the percent with LH >5 at each time point while accounting for repeated measures collected longitudinally on each subject. Time was treated as fixed, AR1 was assumed for the correlation structure, and Fisher’s LSD was used in the pairwise comparison of time points 1 vs 3, 1 vs 6, and 3 vs 6. Correlations of basal and GnRHa-stimulated peak serum LH values were analyzed by Spearman’s rank correlation.

### Subjects

Of the 155 subjects identified with premature sexual development who had undergone a leuprolide stimulation test, 48 were excluded. Thirty eight (38) had inadequate follow-up, 3 were diagnosed with organic disorders of hypothalamic-pituitary axis and 7 had peripheral puberty e.g. congenital adrenal hyperplasia, testotoxicosis and McCune Albright syndrome. Thus the total number of subjects included for analyses were 107. There were 21 girls in Group A with non-progressive puberty with thelarche. Group B, non-progressive puberty with adrenarche, had 15 subjects of which 12 were girls and 3 were boys. Finally, Group C with central progressive puberty included 71 children (58 girls & 13 boys) (Table 1).

**Table 1:** Patient Characteristics

	**Premature Thelarche**	**Premature Adrenarche**	**Central Precocious Puberty**
Patients#	21 (girls)	15 (12 girls, 3 boys)	71 (58 girls, 13 boys)
Chronological Age	5.04 ± 0.46 **	6.0 ± 0.55 *	7.78 ± 0.18
Bone Age	6.73 ± 0.57 **	8.64 ± 0.57	10.46 ± 0.25

Values represent “Mean ± SEM”.

*P <0.05 (compared to puberty group), **P < 0.005 (compared to puberty group).

### Baseline hormone concentrations

#### Group A (Premature thelarche)

Basal serum LH concentrations were 0.1 ± 0.0 mIU/ml. None had a basal LH concentration > 0.1 mIU/mL. Mean basal serum FSH was 2.18 ± 0.3 mIU/ml. All 21 girls had a basal LH/FSH ratio < 1. The basal serum concentrations of estradiol were 0.37 ± 0.15 ng/dL (pre-pubertal <1.5 ng/dL). Serum testosterone was not measured in this group.

#### Group B (Premature adrenarche)

**Figure 1:**
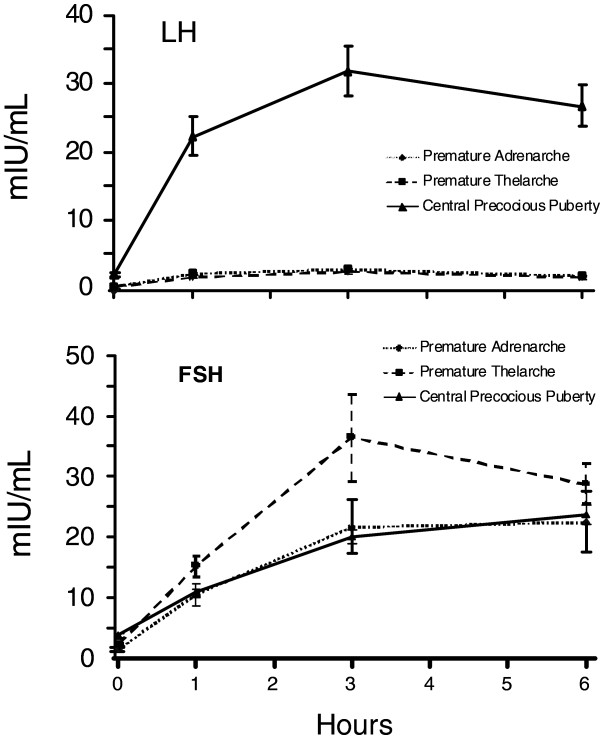
**Baseline and peak stimulated serum LH (upper panel) and FSH (lower panel) concentrations (mean ± SEM) at 1, 3, and 6 hours after leuprolide injection.** LH concentrations in central precocious puberty were significantly higher than in either the children with premature thelarche or premature adrenarche (P< 0.005).

**Figure 2:**
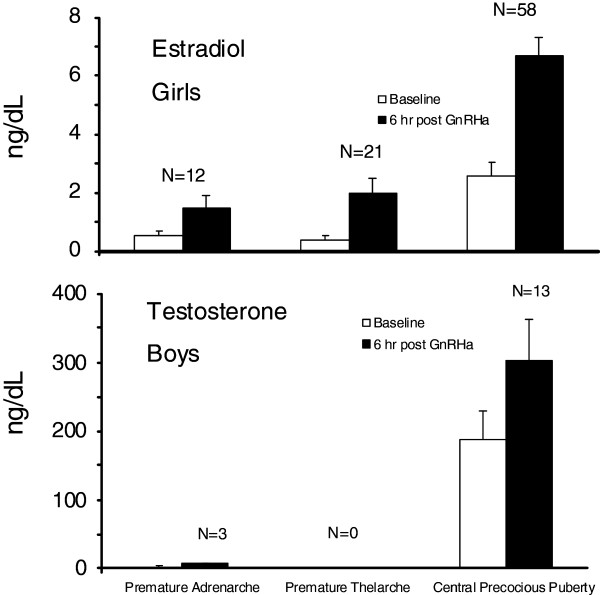
**Baseline and peak stimulated serum Estradiol (upper panel) and Testosterone (lower panel) concentrations (mean ± SEM) at baseline and 6 hours after leuprolide injection.** Estradiol and Testosterone concentrations in central precocious puberty were significantly higher than in either the children with premature thelarche or premature adrenarche (P< 0.005).

Basal serum LH concentrations were 0.1 ± 0.0 mIU/ml whereas basal FSH concentrations were 1.48 ± 0.31 mIU/ml (Figure 1). All 15 subjects with premature adrenarche had a basal LH/FSH ratio < 1. The basal estradiol concentration in the girls (n = 12) was 0.51 ± 0.17 ng/dL. Although the mean basal estradiol concentration was slightly higher than the patients with premature thelarche, it was statistically insignificant (P = 0.38). Additionally, their estradiol levels were in the pre-pubertal range and clinical follow up confirmed the diagnosis of premature adrenarche. Mean testosterone concentrations in the boys (n = 3) was 2.05 ± 0.34 ng/dL (pre-pubertal <10 ng/dL) (Figure 2).

#### Group C (true central precocious puberty)

The basal LH and FSH concentrations were 1.96 ± 0.26 and 3.68 ± 0.31 mIU/mL, respectively (Figure 1). Both were higher than in either the children with premature thelarche or premature adrenarche (p <0.005). Of the 58 female subjects with true puberty, fifteen (26%) had a basal LH value < 0.1 mIU/mL. All 13 of male subjects had a basal LH value > 0.1 mIU/mL (ranged from 0.5-5.3 mIU/mL). Basal estradiol concentrations of the 58 female subjects were 2.59 ± 0.46 ng/dl, and basal testosterone concentrations of the 13 male subjects were 187.69 ± 41.84 ng/dl (Figure 2).

### Leuprolide - Stimulated Hormonal Concentrations

#### Group A (premature thelarche)

**Figure 3:**
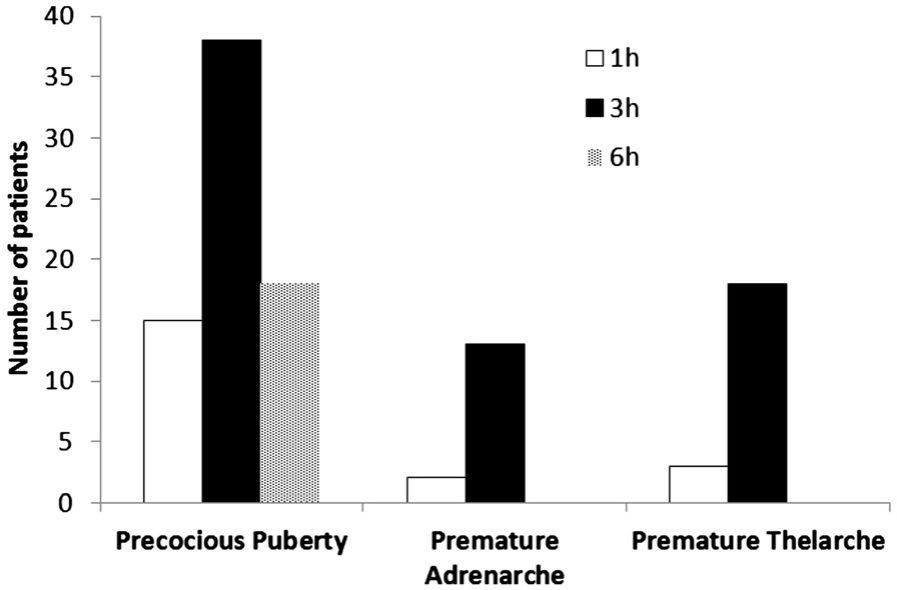
Distribution of number of patients with LH concentration peaked at 1, 3 and 6 hours after leuprolide injection.

All 21 girls with a clinical diagnosis of premature thelarche had a peak stimulated LH concentration < 5 mIU/mL (Figure 1). Of these, 3 had a peak LH concentration at 1 h (14%), 18 at 3 h (86%), 0 at 6 h (Figure 3). Two girls had values at 1 and 3 h which were identical. Plasma FSH concentrations increased and peaked at 3 hr and decreased slightly by 6 h. All of these girls had stimulated LH/FSH ratios of < 1 at 1, 3 and 6 hours. Mean stimulated estradiol concentrations of these 21 girls at 6 hours was 2.00 ± 0.5 ng/dL (Figure 2).

#### Group B (premature adrenarche)

Of the 15 children with premature adrenarche (Figure 1) only 1 girl had a stimulated LH concentration > 5 mIU/mL; the child had a baseline LH = 0.1 mIU/mL and peak stimulated plasma LH concentrations of 5.3 and 5.2 mIU/mL at 3 and 6 h, respectively. Clinically, her diagnosis remained premature adrenarche after 23 months follow up. Among these 15 children, two had a peak stimulated LH at 1 h (13%), 13 at 3 h (87%), none at 6 hr (Figure 3). One child had values of LH that were equal at 1 and 3 h. All had a stimulated LH/FSH ratio < 1 at 1, 3 and 6 hours. It was surprisingly observed that children with premature adrenarche had an FSH-predominant response in our cohort. Their plasma FSH concentrations increased following leuprolide injection and peaked at 3 h but the peak concentrations were significantly less than those of the girls with premature thelarche. We noted that two of the patients in this cohort who were significantly younger (15 months and 3 years) than most patients had a significant FSH–predominant response, but our clinical observation confirmed the diagnosis of premature adrenarche. The mean stimulated serum estradiol of the 12 girls was 1.46 ± 0.42 ng/dl at 6 h and the mean stimulated serum testosterone of the 3 boys was 6.6 ± 1.23 ng/dL at 6 hours (Figure 2).

### Group C (central precocious puberty)

#### Group C Overall Results

Of the 71 children with central precocious puberty (Figure 1) 15 subjects had a maximum plasma concentrations of LH at 1 h (21%), 38 at 3 h (54%), 18 at 6 h (25%) and 2 subjects had a maximum concentration at both 3 & 6 hours (Figure 3). The plasma LH concentrations were higher than in either the children with premature thelarche or premature adrenarche (p < 0.005).

The mean stimulated FSH concentrations of girls with premature thelarche (Group A) were higher than children with central precocious puberty or premature adrenarche at both 1 and 3 h, but not at 6 h. Maximal FSH responses were detected 3 hours post stimulation in children with premature adrenarche and thelarche but at 6 h in children with central precocious puberty (Figure 1).

All 13 boys with central precocious puberty had a stimulated LH/FSH ratio >1 at 1 & 3 hours. In contrast, only 32 (70%) girls with central precocious puberty, and a stimulated LH > 5 mIU/mL, had a stimulated LH/FSH ratio > 1 at 1 and 3 hours.

Mean Stimulated testosterone concentrations of 13 male subjects with central precocious puberty (302 ± 61.3) were higher than the boys with premature adrenarche (6.6 ± 1.2 ng/dl) at 6 h, (P < 0.005). The mean stimulated plasma estradiol concentrations of the 58 girls with central precocious puberty (6.68 ± 0.64 ng/dL) were higher than those of the girls in Groups A and B at 6 h (p < 0.005) (Figure 2).

### Concordant and Discordant Stimulated LH with Clinical Precocious Puberty

Pubertal subjects were divided in two groups based on their responses to leuprolide challenge:

a. With a concordant response for the leuprolide challenge (LH > 5 mIU/mL)

Out of 71 children with clinical evidence of progressive puberty, 59 subjects (83%) had a pubertal response to leuprolide challenge (13 boys and 46 girls). Among these 59 subjects, 52 (88%) had a peak LH > 5 mIU/mL at 1 h (95% CI: 80-96%), but all 59 children (100%) had a peak LH >5 mIU/mL at 3 h (95% CI: 94-100%), P = 0.005 (Figure 3). All 13 boys had clinical evidence of true puberty, and a pubertal response (LH > 5mIU/mL) at 1, 3 and 6 h. Out of these 13 boys, 11 had a maximum concentration of LH at 3 h. Eleven (11) boys (85%) and 32 girls (70%) were treated with GnRHa.

b. With a discordant response for the leuprolide challenge (LH < 5 mIU/mL)

Among 71 children with clinical evidence of true puberty, 12 girls (17%) had a pre-pubertal response (LH < 5 mIU/mL) to Leuprolide challenge despite pubertal progression. Among these 12 subjects, the peak stimulated LH concentrations ranged from 0.9 to 4.6 mIU/mL regardless of the sampling time but had a predominant FSH response, such that we know that the leuprolide was in fact administered. Their stimulated estradiol concentrations at 6 h were not different from groups A and B (data not shown).

#### Diagnostic values of the measured hormones in the evaluation of central precocious puberty

Although not the primary purpose of this study, we evaluated and compared the relative diagnostic values of different parameters including basal LH, estradiol and testosterone, stimulated LH, LH/FSH ratio in the prediction of pubertal status. Our data demonstrates that in boys basal LH (>0.1 mIU/mL), testosterone concentrations (≥10 ng/dL), basal and stimulated LH/FSH ratios (at 1 and 3 h) have excellent sensitivity and specificity all to be 100% (Table 2). However, in girls basal LH > 0.1 mIU/ml, basal and stimulated LH/FSH ratios and basal estradiol (≥1.5 ng/dL) have low sensitivity though excellent specificity (Table 2). When serial potential predictors were combined, the sensitivities were reduced even though specificities were improved (Table 2). Compared to stimulated LH concentration at 1 h, the LH concentration > 5mIU/ml at 3 h had better sensitivity (83% vs 73%) without compromising specificity (97% vs 100%). This cut off also has optimal sensitivity (83%) and specificity (97%) when compared to a lower cut off of 3mIU/ml or a higher cut off of 7mIU/ml (Table 2).

**Table 2:** Diagnostic values of basal LH, Estradiol, Testosterone, stimulated LH and the ratio of LH/FSH for Puberty

**Predictors of puberty**	**Sensitivity**	**Specificity**	**PPV**	**NPV**
Basal LH > 0.1 mIU/ml (M)	100%	100%	100%	100%
Basal LH > 0.1 mIU/ml (F)	67%	100%	100%	63%
Basal Estradiol ≥ 1.5 ng/dL	50%	94%	94%	52%
Basal Testosterone ≥ 10 ng/dL	100%	100%	100%	100%
LH at 1 h ≥ 5 mIU/ml	73%	100%	100%	80%
LH at 3 h ≥ 5 mIU/ml	83%	97%	98%	74%
LH at 3 h ≥ 3 mIU/ml	92%	75%	88%	82%
LH at 3 h ≥ 7 mIU/ml	80%	100%	100%	71%
Basal LH/FSH >1 (M)	100%	100%	100%	100%
Basal LH/FSH >1 (F)	10%	100%	100%	39%
LH/FSH at 1 h >1(M)	100%	100%	100%	100%
LH/FSH at 1 h >1(F)	50%	100%	100%	53%
LH/FSH at 3 h >1(M)	100%	100%	100%	100%
LH/FSH at 3 h >1(F)	45%	100%	100%	51%
LH at 1 h ≥ 5 mIU/ml and basal LH/FSH >1	61%	100%	100%	56%
LH at 3 h ≥ 5 mIU/ml and basal LH/FSH >1	16%	100%	100%	38%
LH at 3 h ≥ 5 mIU/ml and LH/FSH at 1 h >1	59%	100%	100%	56%

M – Male, F- Female; PPV – Positive predictive value; NPV – Negative predictive value.

## Discussion

In the early phase of central sexual precocious puberty, laboratory confirmation is important to provide an accurate diagnosis and appropriate therapy. When random plasma LH concentrations are low in the presence of physical findings suggestive of precocious puberty, GnRHa stimulation testing is recommended to determine activation of hypothalamic-pituitary-gonadal axis. Despite wide utilization of the GnRHa stimulation test, the timing of blood sampling remains controversial if a single sample protocol is used. In our present study, the peak LH response occurred 3 hours post leuprolide stimulation test in those with true central precocious puberty. However, only 59 of 71 of the children with true central precocious puberty had an LH concentration > 5 mIU/mL at 3 hours. When compared to the 1 h value, the 3 h value was higher (p < 0.005) and had better sensitivity in diagnosing central precocious puberty. We recommend that a 3 h sample should be considered for those cases in which clinical presentation and base line laboratory values are not conclusive, despite the practical difficulties posed by a prolonged test protocol, particularly for those families traveling at a distance.

Girls with central precocious puberty in the early phase of activation of the hypothalamic-pituitary-gonadal axis are capable of clinically relevant estradiol production, which may occur in the face of low LH secretion and low LH/FSH ratios [2]. This observation is puzzling and one speculation is that endocrine or paracrine factors other than LH and FSH may play an important role in amplifying the effects of gonadotropins on ovarian E2 secretion in the early phase of sexual precocity [2]. Among our subjects with clinical evidence of precocious puberty, 12 girls with Tanner stage II-III breast development had a pre-pubertal response to leuprolide challenge (LH < 5 mIU/mL), a predominant FSH response and therefore a low LH/FSH ratio. Their laboratory findings were indistinguishable from those of subjects with proven premature thelarche and adrenarche. Interestingly, 10 out of these 12 subjects had both breast and pubic hair development at initial presentation, and only 2 subjects presented with just thelarche. Only one of these 12 girls with discordant response required GnRHa treatment. She was 6.5 y of age at presentation with Tanner III breast and pubic hair, a bone age of 10 y and a normal brain MRI. Her baseline LH was 0.1 mIU/mL, peak stimulated LH 3.9 mIU/mL at 3 h and estradiol concentration of 5.2 ng/dl at 6 h despite continued pubertal development. Therefore, clinical judgment and follow up continues to be of great importance in the evaluation of precocious puberty.

In our study, the basal plasma LH concentration differentiated the pubertal and pre-pubertal boys, without overlap, and is entirely consistent with the findings of Resende et al. in normal male subjects [8]. However, this was not true for the girls. Twenty six percent of the girls ultimately diagnosed with central precocious puberty (Tanner breast stage II-III at presentation) and pubertal responses to leuprolide had basal serum LH concentrations in the pre-pubertal range (LH <0.1 mIU/mL).

Basal LH concentrations in excess of 0.1 mIU/mL were strongly correlated with a pubertal stimulated LH concentrations (>5 mIU/mL at 3 h) in pubertal subjects (r = 0.842, P, 0.0001) (data not shown). This finding is in agreement with others [9]. Thus, a random LH concentration measured by third-generation assays such as immunochemiluminometric assay is a useful tool in screening for central precocious puberty. However, our experience suggests that a GnRHa stimulation test should be considered when a basal serum LH is inconclusive or does not fit with the clinical presentation. This conclusion is further strengthened in that none of our children with either premature thelarche or premature adrenarche had a random serum LH > 0.1 mIU/mL.

Although mean spontaneous serum FSH concentrations were greater in children with central precocious puberty (p < 0.005) and provided fair sensitivity and specificity, subjects in groups A and B had predominant FSH responses to leuprolide challenge and mean stimulated serum FSH concentrations in girls with premature thelarche were higher than pubertal children at both 1 and 3 h (P < 0.005). These observations further strengthen the findings of others [9] that the stimulated FSH is of limited utility in partitioning the children with central precocious puberty from those without central stimulation. We also demonstrate that both basal LH/FSH > 1 and the stimulated LH/FSH ratio >1 at 1 and 3 hours are excellent predictors in diagnosing central precocious puberty in boys. This was not true for girls however (Table 2).

## Conclusion

This is the largest group of children reported who have undergone a 6 h leuprolide acetate stimulation test for the evaluation of central precocious puberty. We conclude that in our study a single basal LH measurement using third-generation assays was adequate to diagnose central precocious puberty in boys. In addition, basal LH is adequate to diagnose central precocious puberty in most but not all girls, indicating the need for GnRHa test when a basal LH is inconclusive. Basal testosterone (measured by LCMS) in conjunction with clinical correlation is diagnostic of central precocious puberty in boys. In contrast, a basal estradiol (measured by LCMS) is helpful in most girls, but not all. However, when a GnRHa stimulation test is undertaken, our data demonstrates that a single sample at 3 h is superior in sensitivity and specificity to that of the 1 h sampling time in diagnosing central precocious puberty in girls, and provides the optimal sample to ascertain a diagnosis of central precocious puberty. Obviously, clinical judgment and follow up continues to be essential in that a quarter of our girls with central precocious puberty had discordant clinical findings with those of either the basal or stimulated LH values.

## Abbreviations

GnRH, Gonadotropin-releasing hormone; LH, Luteinizing hormone; FSH, Follicle stimulating hormone; GnRHa, Gonadotropin-releasing hormone analogue; ICMA, Immunochemiluminometric assay; SQ, Subcutaneous.
